# How pictorial warnings change parents’ purchases of sugar-sweetened beverage for their children: mechanisms of impact

**DOI:** 10.1186/s12966-023-01469-3

**Published:** 2023-06-23

**Authors:** Marissa G. Hall, Anna H. Grummon, Tara Queen, Allison J. Lazard, Isabella C. A. Higgins, Ana Paula C. Richter, Lindsey Smith Taillie

**Affiliations:** 1 Department of Health Behavior, Gillings School of Global Public Health, University of North Carolina at Chapel Hill, Chapel Hill, NC USA; 2 Lineberger Comprehensive Cancer Center, University of North Carolina at Chapel Hill, Chapel Hill, NC USA; 3 Carolina Population Center, University of North Carolina at Chapel Hill, Chapel Hill, NC USA; 4 Department of Nutrition, Harvard TH Chan School of Public Health, Boston, MA USA; 5 Department of Population Medicine, Harvard Medical School, Harvard Pilgrim Health Care Institute, Boston, MA USA; 6 Department of Pediatrics, Stanford University School of Medicine, Palo Alto, CA USA; 7 Hussman School of Journalism and Media, University of North Carolina at Chapel Hill, Chapel Hill, NC USA; 8 Department of Nutrition, Gillings School of Global Public Health, University of North Carolina at Chapel Hill, Chapel Hill, NC USA

**Keywords:** Sugar-sweetened beverages, Pictorial warnings, Warning labels, Mediators, Childhood obesity

## Abstract

**Background:**

Pictorial health warnings on sugar-sweetened beverages (SSBs) are a promising policy for preventing diet-related disease in children. A recent study found that pictorial warnings reduced parents’ purchases of SSBs for their children by 17%. However, the psychological mechanisms through which warnings affect parental behavior remain unknown. We aimed to identify the mechanisms that explain how pictorial warnings affect parents’ SSB purchasing behavior for their children using secondary data from a randomized trial.

**Methods:**

In 2020–2021, parents of children ages 2 to 12 years (*n* = 325) completed a shopping task in a convenience store laboratory in North Carolina, USA. Participants were randomly assigned to a pictorial warnings arm (SSBs displayed pictorial health warnings about type 2 diabetes and heart damage) or a control arm (SSBs displayed a barcode label). Parents then bought a beverage for their child and took a survey measuring 11 potential psychological mediators, selected based on health behavior theories and a model explaining the impact of tobacco warnings. We conducted simple mediation analyses to identify which of the 11 mechanisms mediated the impact of exposure to pictorial warnings on purchasing any SSBs for their children.

**Results:**

Two of the 11 constructs were statistically significant mediators. First, the impact of pictorial warnings on the likelihood of purchasing any SSB was mediated by parents’ perceptions that SSBs were healthier for their child (mediated effect= −0.17; 95% CI = − 0.33, − 0.05). Second, parents’ intentions to serve SSBs to their children also mediated the effect of warnings on likelihood of purchasing any SSB (mediated effect= −0.07, 95% CI=-0.21, − 0.003).

**Conclusions:**

Pictorial warnings reduced parents’ purchases of SSBs for their children by making parents think SSBs are less healthful for their children and reducing their intentions to serve SSBs to their children. Communication approaches that target healthfulness perceptions and intentions to serve SSBs may motivate parents to buy fewer SSBs for their children.

**Supplementary Information:**

The online version contains supplementary material available at 10.1186/s12966-023-01469-3.

## Introduction

Consumption of sugar-sweetened beverages (SSBs) is associated with numerous health problems in children, including obesity and dental caries [1, 2]. Parents have a large influence on the types of foods and drinks that children consume [3, 4]. Informing parents about the health harms associated with SSBs is therefore a promising strategy for reducing children’s SSB consumption. One policy that could help inform parents and reduce SSB purchases is requiring SSB containers to display warning labels [5, 6]. Since 2011, nine US jurisdictions have proposed legislation requiring that warnings stating the health consequences of SSBs be displayed on SSB containers, advertisements, or at the point of sale [7]. Globally, 10 countries have passed laws to require nutrient warnings on foods and beverages that exceed thresholds for nutrients of concern, including warnings about high sugar content in SSBs [8].

Mounting research indicates that SSB warnings are a promising tool for reducing parents’ selection of SSBs for their children. Three experiments have found that warnings on SSBs reduced parents’ hypothetical selection [9, 10] and purchasing of SSBs [11] for their children. However, the mechanisms explaining the impact of SSB warnings on parents’ behavior remain unclear. Understanding these mechanisms could suggest actionable strategies for designing SSB warnings that can more effectively reduce parents’ purchases of SSBs for their children. For example, if SSB warnings reduce parents’ purchases by heightening attention to the warnings, policymakers should design warnings with the goal of attracting as much attention as possible. Identifying the mechanisms of impact among parents can also inform the design of other communication approaches to reduce children’s SSB consumption, such as mass media campaigns.

Health behavior theories and research suggest several potential mechanisms of SSB warnings’ impact. First, warnings could change behavior by eliciting *message reactions* – that is, parents’ immediate processing of the message in their head or body. For example, warnings could change behavior by grabbing parents’ attention, causing them to feel negative emotions such as fear, or by prompting them to think about the harms of using a product. Multiple theories provide support for these reactions being mechanisms of behavior change, including the Elaboration Likelihood Model [12] and the Extended Parallel Process Model [13]. Other potential mediators of SSB warnings’ impacts include *attitudes and beliefs* about SSBs. Theories such as the Health Belief Model [14, 15] and Theory of Planned Behavior [16] posit that attitudes and beliefs could be powerful mechanisms of behavior change. For example, SSB warnings could change parents’ purchase behaviors by changing their perceptions of healthfulness of SSBs for their children and perceived likelihood of SSBs causing health problems in children. Finally, the Theory of Planned Behavior [16] and Theory of Reasoned Action [17] posit that behavioral *intentions* are mediators of behavior change [16], a finding that has been supported by prior mediation studies of warnings [18, 19].

Two studies have examined how warnings change behavior among adults shopping for themselves, finding that SSB warnings reduced SSB purchasing or selection primarily by heightening message reactions [19, 20]. Similar to the studies with adults making decisions for themselves, one study of parents found that negative emotional reactions mediated the impact of warnings on parents’ hypothetical selection of SSBs for their children [10]. However, a second study of parents’ hypothetical beverage purchases for their children found that warnings worked by making parents think SSBs were less healthy [21], a construct that typically does not play a role in how warnings affect general adult populations. It is possible that healthfulness perceptions are more important for parents acting on behalf of their children than for adults shopping for themselves because parents rate nutritional quality as the most important factor they consider when selecting foods for their children [22]. Although initial studies of mediators with parents are suggestive, these studies did not assess parents’ actual purchase behaviors. Studies with objective purchasing outcomes are necessary to provide a more externally valid evaluation of mediators of SSB warnings’ effects among parents. To fill this gap, this study aimed to examine mediators of the impact of pictorial SSB health warnings on parents’ purchases of SSBs for their children.

## Methods

### Participants

The current study used secondary data from a randomized trial with 326 parents of children ages 2–12 years old [11]. From January to March 2020, we recruited trial participants from Central North Carolina through in-person recruitment, flyers, email listservs, Craigslist ads, Facebook ads, and word of mouth. To mask the purpose of the trial, all study materials stated that the study sought to understand the factors that affect consumers’ purchasing decisions in a convenience store environment. Due to COVID-19, we paused recruitment and enrollment beginning in March 2020, and resumed recruitment in October 2020 after implementing a COVID-19 safety protocol. Study enrollment was completed in March 2021.

To be eligible, participants had to be at least 18 years of age and the parent or guardian (hereafter “parent”) of at least one child ages 2–12 years old who consumed at least one SSB in the past week. Additionally, participants had to be able to read and speak English or Spanish, use a tablet or computer to take a survey, and attend one in-person study visit. The University of North Carolina Institutional Review Board approved the study (IRB #19–0277) and participants provided written informed consent. All study materials were available in English and Spanish.

### Setting

The study took place at the UNC Mini Mart, a 245-square-foot convenience store designed for research purposes, in Chapel Hill, NC [23]. The Mini Mart contains a commercial refrigerator, gondola shelving units, and a check-out stand with a point-of-sale system. We stocked the Mini Mart with 33 types of single-serving beverages, more than 130 types of food items, and 31 household good items. To determine which beverages to stock, we used 2014 Nielsen Homescan Data to examine top selling beverages at convenience stores in the US among households with children in each of 6 beverage categories: fruit-flavored drinks, sodas, flavored milks, sports and energy drinks, flavored waters, and sweet teas. For every SSB sold, there was a comparable non-sugary option displayed next to the SSB in the refrigerator. All SSBs and their non-sugary equivalents were sold for the same price. A validation study with parents found that nearly all participants reported that their Mini Mart purchases were similar to their typical purchases (96%), the Mini Mart felt like a real store (94%), and they could imagine doing their shopping in the Mini Mart (92%) [23]. Rates of SSB purchasing were similar in the store as compared to real-world purchases measured via receipts [23].

### Procedures

**Fig. 1:**
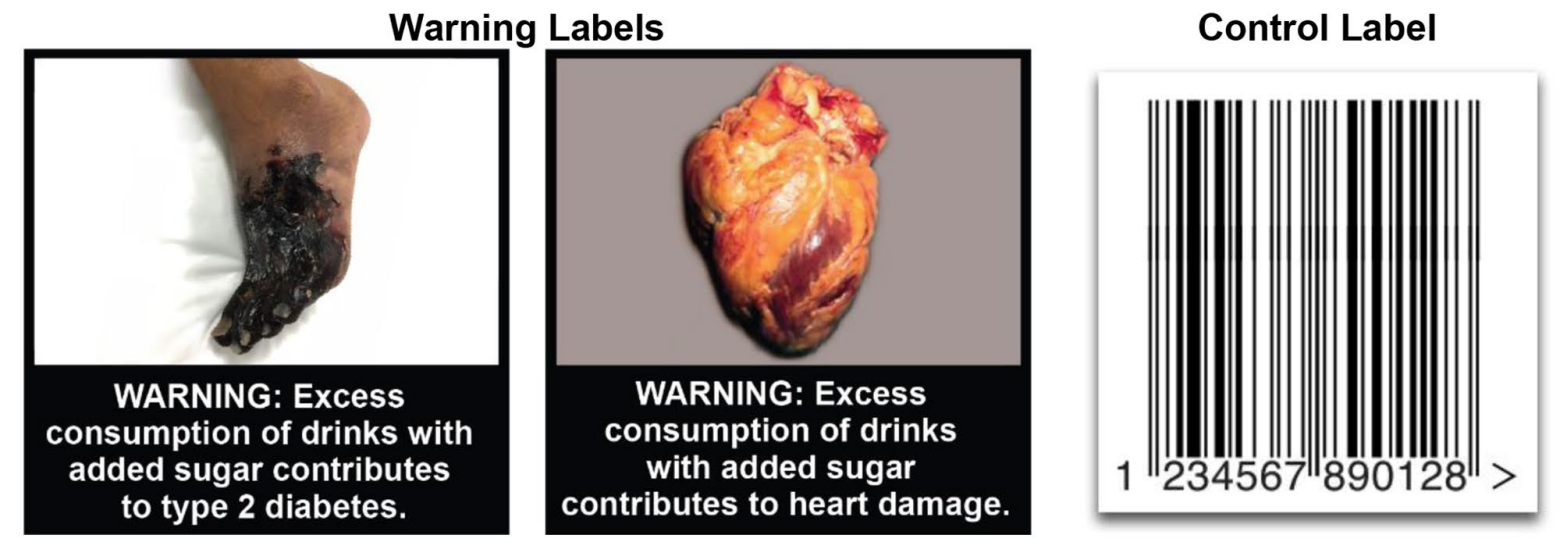
Warning and control labels used in a trial of pictorial health warnings for sugar-sweetened beverages

The trial used a parallel arm study design, with staff randomly assigning participants to one of the two trial arms: pictorial warnings or control labels. Staff prepared the Mini Mart before a participant’s arrival based on the assigned trial arm. In the pictorial warnings arm, staff applied one of two warning labels (Fig. 1) to the front of all SSBs in the Mini Mart. The two pictorial warnings read “WARNING: Excess consumption of drinks with added sugar contributes to type 2 diabetes” and “WARNING: Excess consumption of drinks with added sugar contributes to heart damage” and were accompanied by photographs representing each of the topics. As reported previously [24], we developed the warnings through a multiphase process with a professional designer, a stakeholder advisory board, and two rounds of quantitative pre-testing. About half of the SSBs in the Mini Mart displayed the heart damage warning label, and the other half displayed the type 2 diabetes label. In the control arm, staff applied a neutral barcode label to all SSBs to control for the presence of a study label and for the amount of branding obscured by the label.

Before participants entered the store, staff instructed them to select one snack and one beverage for their child, as well as one household item. This shopping task was designed to mask the purpose of the study. Research staff informed the participants that one of the items would be randomly selected at the checkout counter for the participant to take home. After the shopping task, participants completed a survey programmed in Qualtrics on a computer or tablet in a separate room. Participants received the beverage and cash for a total value of $40 for their participation in the study.

### Measures

In the current study, we examined mediators of the impact of the pictorial warnings on purchasing any SSBs in the Mini Mart (yes/no), which was the primary outcome for both this study and the main trial [11]. We also examined mediation of the impact of pictorial warnings on SSB calories purchased in the Mini Mart as a secondary outcome, for comparability with a similar mediation study with text-only warnings on SSBs [19].

The survey assessed a range of potential psychological mediators using measures adapted or used verbatim from previous studies (Supplementary Table 1). We examined three categories of potential mediators: message reactions, attitudes and beliefs, and intentions, drawing on research from prior SSB warning studies [5, 6, 25, 26], as well as health communication and behavior theories [12, 13, 27, 28].

First, the survey assessed three different *message reactions*: attention to the labels, negative emotional reactions, and thinking about the harms of SSBs. Second, the survey assessed six types of *attitudes and beliefs*, including perceived amount of added sugar in SSBs, perceived healthfulness of SSBs for their child, appeal of SSBs for their child, perceived tastiness of SSBs for their child, perceived likelihood of child experiencing health problems due to SSBs, and injunctive norms to limit SSBs for their child (i.e., the perception that other people want them to limit SSBs for their child). Finally, the survey assessed two types of *intentions*: anticipated social interactions (i.e., intentions to talk to others about the study labels) and intentions to serve SSBs to their child in the next week. All of the mediators used response scales ranging from low values coded as 1 to high values coded as 5, except for intentions to serve SSBs to their child, which ranged from 0 times to 21 times per week.

### Analysis

**Fig. 2:**
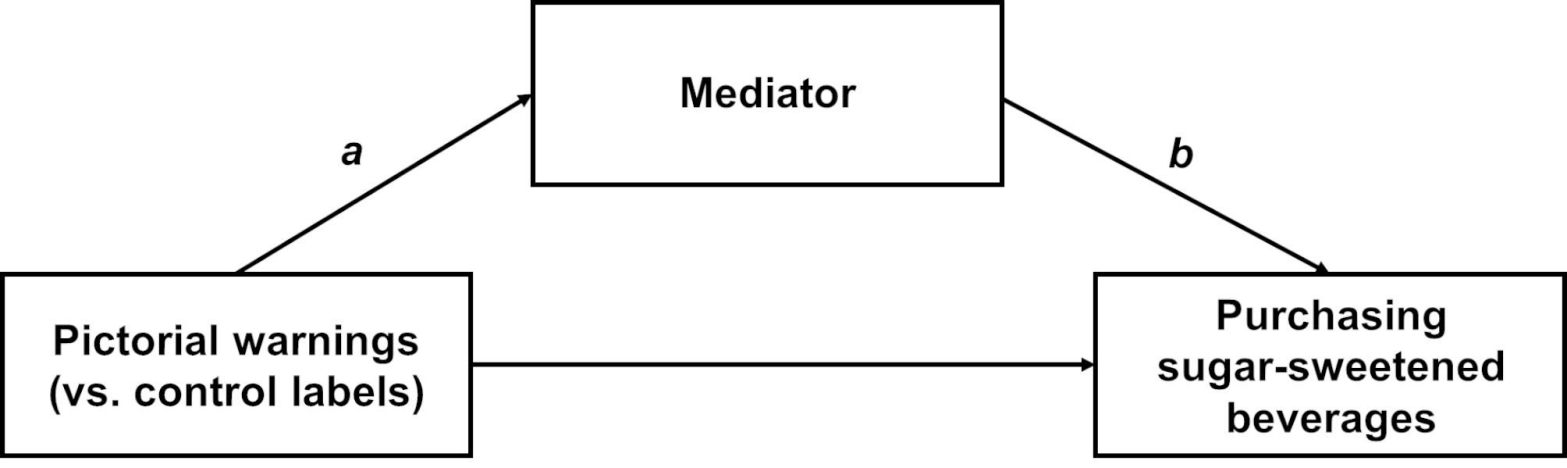
Primary mediation model

The analytic sample included 325 participants with complete data on the primary outcome in the main trial, excluding one person with missing data on the primary outcome of purchasing any SSB. Mediation analyses used the MacKinnon approach [29], assessing the impact of trial arm on the mediator (*a* pathway), the association of the mediator with the outcome while controlling for trial arm (*b* pathway), and the product of the a-pathway and the b-pathway (i.e., the indirect or mediated effect, a*b; Fig. 2). We opted to test single mediator models as a first step in understanding the independent effects of each potential mechanism and to avoid potential collinearity among a large number of mediators with some conceptual overlap.

Analyses used the PROCESS macro for SPSS (version 4.1) [30] using bootstrapped 95% confidence intervals with 10,000 repetitions; this approach does not assume that indirect effects are normally distributed [31]. We repeated these models for each of the two outcomes (purchase of any SSB and calories purchased from SSBs). All mediators were treated as linear variables in modeling since they were all measured continuously. We determined that mediation had occurred if the 95% confidence interval around the indirect effect did not cross zero. Models of purchasing any SSB were estimated using logistic regression; models of calories purchased from SSBs were estimated using OLS regression. As there were limited missing data on the mediators (0–17 observations depending on variable; no more than 5% participants had missing data on any of the mediators), we used complete case analyses for each model. We calculated the proportion of the total effect mediated for each significant mediator for the models with calories purchased from SSBs as the outcome (indirect effect/total effect). We did not calculate the proportion of the total effect mediated for the effect of warnings on parents’ likelihood of purchasing any SSBs because the PROCESS macro does not estimate the total effect for dichotomous outcomes.

We planned not to adjust for covariates in based on CONSORT 2010 guidance for RCTs recommending that adjustment is only needed for variables with strong prognostic strength (e.g., stratification variables) [32]. However, we performed sensitivity analyses to explore whether adjustment might be warranted for the mediation analyses. We first examined the correlation between key demographics (i.e., age, ethnicity, educational attainment, and annual household income) and the primary outcome of purchasing any SSBs. We planned to re-run the mediation analyses controlling for any characteristics that were significantly correlated with the primary outcome; however, there were no significant correlations, so the mediation models did not include any covariates.

## Results

Parents’ mean age was 38 years, and most (77%) were women (Table 1). Slightly fewer than half (45%) were non-Hispanic white, 25% were non-Hispanic Black, and 20% were Latino/a. About half (55%) of participants had an annual household income under $50,000, and 42% had a high school diploma or less education. About a third (38%) of participants shopped for a child ages 2–5 years, and 62% shopped for a child ages 6–12 years.

**Table 1:** Participant characteristics (*n* = 325 parents)

Characteristic	Control arm		Pictorial warnings arm	
	*n*	%	*n*	%
Age, in years				
18–29	24	15%	21	13%
30–39	65	40%	74	45%
40–49	53	33%	54	33%
50+	20	12%	14	9%
Mean (SD)	38.9	8.3	37.8	7.8
Gender				
Man	41	25%	32	20%
Woman	120	74%	130	80%
Another gender identity	1	1%	1	1%
Sexual orientation				
Straight or heterosexual	148	94%	145	90%
Gay, lesbian, bisexual, or homosexual	6	4%	14	9%
Another sexual orientation	4	3%	3	2%
Race and ethnicity				
Non-Hispanic white	72	46%	70	44%
Hispanic white	9	6%	10	6%
Hispanic, no race reported	14	9%	15	9%
Hispanic, other race(s)	8	5%	9	6%
Non-Hispanic Black or African American	46	29%	34	21%
Non-Hispanic Asian	6	4%	7	4%
Non-Hispanic, more than one race	3	2%	13	8%
Non-Hispanic, other race			2	1%
Educational attainment				
Less than high school diploma or GED	11	7%	15	9%
High school diploma or GED	55	35%	55	34%
Four-year college degree	42	27%	46	29%
Master’s degree, graduate degree, or more	47	30%	44	28%
Annual household income				
$0-$24,999	49	30%	50	32%
$25,000-$49,999	39	24%	41	26%
$50,000-$74,999	16	10%	18	11%
$75,000+	58	36%	49	31%
Number of people in household, mean (SD)	3.6	1.2	3.6	1.3
Body-mass index (kg/m^2^)				
<18.5	6	4%	4	3%
18.5 to < 25	43	29%	43	28%
25 to < 30	43	29%	45	30%
≥30	57	38%	60	39%
Mean (SD)	29.7	9.9	29.3	8.0
Nutrition Facts Panel use				
Never or rarely	26	16%	25	16%
Sometimes	46	29%	49	30%
Often or all the time	89	55%	87	54%
Frequency of needing help reading medical information				
Never	130	81%	132	81%
Sometimes	23	14%	18	11%
Often/Always	7	4%	12	7%
Language of survey administration				
English	142	88%	140	86%
Spanish	20	12%	23	14%
Reads and speaks…				
Mostly or only English	132	81%	130	80%
Spanish and English equally	10	6%	14	9%
Mostly or only Spanish	20	12%	19	12%
Age of child the parent shopped for, in years				
2–5	61	38%	63	39%
6–12	101	62%	100	61%
Mean (SD)	7.3	3.4	7.1	3.3
Gender of child the parent shopped for				
Boy	72	44%	75	46%
Girl	88	54%	88	54%
Another gender identity	2	1%		
Child consumed sugar-sweetened beverage 1/wk or more over past 30 days (not mutually exclusive)				
Soda	68	42%	58	36%
Sports drinks	50	31%	50	31%
Flavored water	43	27%	38	24%
Fruit drink	102	64%	95	59%
Flavored milk	102	65%	98	61%
Sweetened coffee or tea	41	26%	35	22%
Time of participation				
Pre-COVID-19 pandemic	64	40%	65	40%
During COVID-19 pandemic	98	60%	98	60%

*Note*. SD, standard deviation. Missing demographic data ranged from 0–7%. Characteristics did not differ by trial arm (all *p* > .05)

### Impact of pictorial warnings on mediators

Pictorial warnings influenced 8 of the 11 hypothesized mediators (*a* pathway), as reported previously [11] (Table 2). Pictorial warnings changed all three message reactions, leading to greater attention, negative emotional reactions, and thinking about the harms of drinking SSBs (all *p* < .001). Pictorial warnings changed two of six attitudes and beliefs: warnings led to lower perceptions that SSBs are healthy for their child (p < .01) as well as stronger injunctive norms to limit serving SSBs to their child (*p* = .01). Pictorial warnings did not elicit changes in perceived amount of added sugar in SSBs, appeal of SSBs for child, perceived tastiness of SSBs for child, or perceived likelihood of child having health problems due to SSBs (all *p* > .05). Finally, pictorial warnings led to greater anticipated social interactions and lower intentions to serve SSBs to their child (both *p* < .05).

**Table 2:** Mediation of pictorial health warnings’ effect on parents’ likelihood of purchasing any sugar-sweetened beverage for their children

Mediator		Warnings on mediator	Mediator on purchasing	Total effect	Mediated effect
	*n*	*a*	*b*	*c*	*a x b* (95% CI)
**Message reactions**					
Attention to the labels	322	**1.48 (1.21, 1.76)**	0.13 (-0.06, 0.31)	**− 0.93 (-1.48, -0.38)**	0.19 (-0.08, 0.49)
Negative emotional reactions	320	**1.87 (1.66, 2.09)**	-0.10 (-0.34, 0.14)	**-0.53 (-1.17, 0.11)**	-0.19 (-0.67, 0.28)
Thinking about harms of drinking SSBs	322	**2.31 (2.07, 2.55)**	-0.14 (-0.34, 0.07)	-0.42 (-1.09, 0.24)	-0.32 (-0.85, 0.18)
**Attitudes and beliefs**					
Perceived amount of added sugar in SSBs	321	0.10 (-0.06, 0.26)	-0.23 (-0.55, 0.09)	**-0.73 (-1.20, -0.26)**	-0.02 (-0.09, 0.02)
Perceived healthfulness of SSBs for child	321	**-0.26 (-0.44, -0.08)**	**0.64 (0.34, 0.94)**	**-0.60 (-1.08, -0.12)**	**-0.17 (-0.33, -0.05)**
Appeal of SSBs for child	322	-0.08 (-0.30, 0.13)	-0.00 (-0.24, 0.23)	**-0.74 (-1.20, -0.27)**	0.00 (-0.03, 0.04)
Perceived tastiness of SSBs for child	321	-0.17 (-0.37, 0.03)	-0.01 (-0.26, 0.24)	**-0.72 (01.19, -0.26)**	0.00 (-0.05, 0.06)
Perceived likelihood of health problems	309	0.12 (-0.10, 0.33)	**-0.40 (-0.65, -0.15)**	**-0.83 (-1.31, -0.35)**	-0.05 (-0.14, 0.05)
Injunctive norms to limit SSBs for child	316	**0.39 (0.11, 0.67)**	0.10 (-0.09, 0.28)	**-0.81 (-1.29, -0.34)**	0.04 (-0.04, 0.13)
**Intentions**					
Anticipated social interactions	322	**1.73 (1.45, 2.01)**	-0.02 (-0.20, 0.16)	**-0.71 (-1.27, -0.15)**	-0.03 (-0.34, 0.29)
Intentions to serve SSBs to child	320	**-0.35 (-0.64, -0.05)**	**0.20 (0.03, 0.38)**	**-0.65 (-1.12, -0.18)**	**-0.07 (-0.21, -0.003)**

*Note.* SSB = sugar-sweetened beverages. The second column reports coefficients for the *a* pathway, regressing the mediator on treatment arm. The third column shows coefficients for the *b* pathway, regressing purchasing SSBs (yes/no) on the mediator, controlling for treatment arm. Coefficients for both pathways are presented in log odds metric. The final column shows mediated effects (*a × b*). The c pathway represents the coefficient from regressing the outcome on treatment arm; this coefficient may vary due to differing sample sizes. Bold indicates statistical significance, *p* < .05. Confidence intervals for mediated effects were bootstrapped

### Association of mediators on purchasing SSBs

When examining the associations between mediators and purchasing any SSBs, controlling for trial arm (*b* pathway), parents’ perceptions that SSBs were healthier for their children were associated with higher likelihood of purchasing SSBs (*p* < .001). Higher perceived likelihood of child having health problems due to SSBs was associated with a lower likelihood of purchasing SSBs (*p* < .05). Finally, higher intentions to serve SSBs to one’s child was associated with a greater likelihood of purchasing (*p* < .05).

### Mediation

Two of the 11 potential mechanisms were significant mediators of the impact of SSB warnings on parents’ likelihood of buying any SSB for their child (Table 2). Perceived healthfulness of SSBs mediated the effect: pictorial warnings led to lower perceptions that SSBs are healthy for their child (*a* pathway=-0.26), which in turn was associated with a lower likelihood of purchasing of SSBs (*b* pathway = 0.64), resulting in a mediated effect of − 0.17 (95% CI = − 0.33, − 0.05). Intentions to serve SSBs also mediated the effect, such that pictorial warnings led to lower intentions to serve SSBs to their child (*a* pathway=−0.35), which in turn was associated with a lower likelihood of purchasing SSBs (*b* pathway = 0.20), resulting in a mediated effect of − 0.07 (95% CI= −0.21, − 0.003). When examining mediators of the impact of SSB warnings on calories purchased from SSBs (our secondary outcome), perceived healthfulness and intentions to serve SSBs were the only two statistically significant mediators, following the same pattern as for purchasing any SSBs (Table 3). Perceived healthfulness mediated 22% of the total effect of warnings on calories purchased, whereas intentions to serve SSBs mediated 9% of the total effect.

**Table 3:** Mediation of pictorial health warnings’ effect on calories from SSBs purchased by parents for their children

		Warnings on mediator	Mediator on calories	Direct effect	Total effect	Mediated effect
	*n*	*a*	*b*	*c'*	*c*	*a x b* (95% CI)
**Message reactions**						
Attention to the labels	322	**1.48 (1.21, 1.76)**	3.86 (-4.16, 11.89)	**-36.04 (-59.54, -12.54)**	**-30.31 (-50.56, -10.05)**	5.73 (-5.85, 17.47)
Negative emotional reactions	320	**1.87 (1.66, 2.09)**	-2.80 (-13.24, 7.63)	-24.44 (-52.68, 3.81)	**-29.69 (-50.05, -9.33)**	-5.25 (-23.82, 14.49)
Thinking about harms of drinking SSBs	322	**2.31 (2.07, 2.55)**	-6.90 (-16.07, 2.22)	-14.32 (-43.56, 14.93)	**-30.31 (-50.56, -10.06)**	-15.99 (-36.93, 4.71)
**Attitudes and beliefs**						
Perceived amount of added sugar in SSBs	321	0.10 (-0.06, 0.26)	-8.29 (-22.31, 5.72)	**-30.00 (-50.33, -9.67)**	**-30.82 (-51.11, -10.53)**	-0.83 (-3.73, 0.93)
Perceived healthfulness of SSBs for child	321	**-0.26 (-0.44, -0.08)**	**25.48 (13.21, 37.75)**	**-23.31 (-43.40, -3.23)**	**-29.99 (-50.30, -9.68)**	**-6.68 (-13.11, -1.74)**
Appeal of SSBs for child	322	-0.08 (-0.30, 0.13)	-1.92 (-12.40, 8.56)	**-30.47 (-50.77, -10.17)**	**-30.31 (-50.56, -10.06)**	0.16 (-1.14, 1.96)
Perceived tastiness of SSBs for child	321	-0.17 (-0.37, 0.03)	-2.18 (-13.23, 8.86)	**-29.75 (-50.10, -9.40)**	**-29.38 (-49.62, -9.15)**	0.37 (-1.49, 2.83)
Perceived likelihood having health problems	309	0.12 (-0.10, 0.33)	**-13.31 (-23.95, -2.68)**	**-33.81 (-54.20, -13.42)**	**-35.36 (-55.88, -14.84)**	-1.55 (-4.86, 1.57)
Injunctive norms to limit SSBs for child	316	**0.39 (0.11, 0.67)**	4.42 (-3.65, 12.50)	**-34.21 (-54.85, -13.58)**	**-32.47 (-52.86, -12.08)**	1.75 (-1.35, 5.59)
**Intentions**						
Anticipated social interactions	322	**1.73 (1.45, 2.01)**	-1.51 (-9.45, 6.43)	**-27.69 (-52.21, -3.19)**	**-30.31 (-50.56, -10.06)**	-2.61 (-16.30, 11.17)
Intentions to serve SSBs to child	320	**-0.35 (-0.64, -0.05)**	**7.69 (0.17, 15.21)**	**-27.02 (-47.45, -6.58)**	**-29.69 (-50.05, -9.33)**	-2.67 (-7.63, 0.01)

*Note.* SSB = sugar-sweetened beverages. The *a* pathways are reported in Table 2. The *b* pathway represents the coefficient from regressing the outcome on the mediator, controlling for treatment arm. The final column shows mediated effects (*a × b*). The *c* pathway represents the coefficient from regressing the outcome on treatment arm; this coefficient may vary due to differing sample sizes. The *c' *pathway is equiavalent to the indirect effect subtracted from the total effect. Bold indicates statistical significance, *p* < .05

## Discussion

In this study, we found that pictorial health warnings reduced parents’ purchases of SSBs for their children by reducing the perceived healthfulness of SSBs. Additionally, pictorial warnings changed parents’ purchase behavior by lowering their intentions to serve SSBs to their children, in line with health behavior theories (e.g., the Theory of Planned Behavior) that posit that behavioral intentions predict behavior change [16, 17].

We found that perceived healthfulness of SSBs partially explained how SSB warnings reduced parents’ likelihood of purchasing an SSB for their child. SSB warnings led to lower perceptions that SSBs are healthy for their child, which in turn was associated with a lower likelihood of purchasing of SSBs. Additionally, higher perceived likelihood that SSBs could lead to health problems for their child was associated with a lower likelihood of parents purchasing SSBs (though perceived disease likelihood was not a significant mediator). These findings are in line with a prior study that found that parents’ healthfulness and risk perceptions mediated the impact of health warnings on hypothetical selection of SSBs for their children [21]. Together, these findings suggest that communications approaches about SSBs directed toward parents (including warnings) may be more effective if they focus on SSBs’ poor nutritional quality and the health harms associated with overconsuming these products. Warnings may be especially effective for changing beliefs about some SSB types, such as fruit drinks, that are frequently marketed to appear healthful [33–36] and that bear marketing claims known to cause parents to hold incorrect beliefs about the products’ nutritional quality [37].

In our study, message reactions did not mediate the impact of SSB warnings on parents’ purchases of SSBs. These results stand in contrast to two prior studies with adults, finding that message reactions including emotions and thinking about harms, explained how SSB warnings affected adults’ purchase behaviors and intentions [19, 20]. The findings also stand in contrast with one study among parents finding that negative emotional reactions mediated the impact of SSB warnings on selection of SSBs for their child [10]. The results in the present study also contrast with studies finding that message reactions are key mediators of the impact of tobacco warnings on adults’ tobacco-related intentions and behavior [18, 38–42]. In the current study, SSB warnings affected all three message reactions including attention, negative emotions, and thinking about the risks of SSBs, but these changes did not influence parents’ selection of SSBs. Our finding that healthfulness perceptions explained how warnings affected parents also differs from prior studies of mediators underlying both SSB and cigarette warnings’ impacts in general adult populations [18, 19].

The differences in mediators between prior studies of adults and the present study of parents suggest that that the process of reacting to health messages might function differently when making purchasing decisions for oneself, compared to when making decisions about another person (perhaps especially when that person is one’s child). One possible explanation for these differences is that message reactions are processes that reflect an individual’s thoughts and feelings, but do not involve considerations for others. Another possibility is that parents are less informed about what beverages are healthy for their children than for themselves, giving warnings more room to changing healthfulness perceptions. Consistent with this hypothesis, prior research shows that parents tend to believe certain types of SSBs including fruit drinks and sports drinks are healthy options for their children [21, 35, 43] and that warnings can help correct misperceptions about these beverages [21]. Whatever the explanation, the differences in mediation patterns among adults buying products for themselves [19, 20] and parents buying for their children in the current study indicates that future studies should examine mediation patterns separately for these two populations.

Overall, we found many small effects in our models, which mirrors prior research that health communication interventions often lead to a small (~ 5%) change in a desired outcomes [44]. In terms of the mediated effects, we found that perceived healthfulness mediated 22% of the total effect of warnings on calories purchased, whereas intentions to serve SSBs mediated 9% of the total effect. These findings suggest that other unmeasured mechanisms of influence could be acting as important mediators; future studies should examine a broader range of mediators.

Strengths of this study include the randomized controlled design. We also assessed an objective purchasing outcome in the context of naturalistic experimental store setting with a wide variety of real products. Limitations include that participants had only one exposure to the warning labels and mediators and outcomes were measured at only one timepoint. Additional studies should establish patterns of mediation over a longer time period and explore serial mediation using longitudinal data. Finally, it is possible that our surveys did not measure all possible mediators of warning labels; future studies including qualitative research could shed light on potential psychological mediators not assessed in this study.

## Conclusions

This randomized trial found that pictorial SSB warnings reduced parents’ purchases of SSBs for their children by making parents think SSBs are less healthful for their children and changing parents’ intentions about serving SSBs. These results stand in contrast to prior studies showing that message reactions explain the impact of warnings on adults’ SSB purchases for themselves, and suggest that different mechanisms may underly warning effects for parents purchasing for their children compared to adults purchasing drinks for themselves. Warnings and other communications approaches targeting healthfulness perceptions and intentions may be particularly effective for reducing parents’ purchases of SSBs for their children.

## Electronic supplementary material

Below is the link to the electronic supplementary material.

Supplementary Material 1

Supplementary Material 2

Supplementary Material 3

Supplementary Material 4

## Data Availability

The dataset and syntax used in the current study are available from the corresponding author on reasonable request.

## Abbreviations

SSB: Sugar-sweetened beverage
