# Supplementary material for: How pictorial warnings change parents’ purchases of sugar-sweetened beverage for their children: mechanisms of impact
Source: Int J Behav Nutr Phys Act. 2023 Jun 23;20:76. doi: 10.1186/s12966-023-01469-3 (PMC10290296; doi:10.1186/s12966-023-01469-3)
Supplement: Supplementary file 4 — Supplementary Material 4 [file 12966_2023_1469_MOESM4_ESM.docx]

**Supplementary Table**

**How pictorial warnings change parents’ purchases of sugar-sweetened beverage for their children: Mechanisms of impact**

Marissa G. Hall^1,2,3^

Anna H. Grummon^4,5,6^

Tara Queen^1^

Allison J. Lazard^7,2^

Isabella C. A. Higgins^1,3^

Ana Paula C. Richter^1,3^

Lindsey Smith Taillie^8,3^

1. Department of Health Behavior, Gillings School of Global Public Health, University of North Carolina at Chapel Hill, Chapel Hill, NC
2. Lineberger Comprehensive Cancer Center, University of North Carolina at Chapel Hill, Chapel Hill, NC
3. Carolina Population Center, University of North Carolina at Chapel Hill, Chapel Hill, NC
4. Department of Nutrition, Harvard TH Chan School of Public Health, Boston, MA
5. Department of Population Medicine, Harvard Medical School and Harvard Pilgrim Health Care Institute, Boston, MA
6. Department of Pediatrics, Stanford University School of Medicine, Palo Alto, CA
7. Hussman School of Journalism and Media, University of North Carolina at Chapel Hill, Chapel Hill, NC
8. Department of Nutrition, Gillings School of Global Public Health, University of North Carolina at Chapel Hill, Chapel Hill, NC

**Corresponding author:**

Marissa G. Hall, PhD

Assistant Professor

Department of Health Behavior

UNC Gillings School of Global Public Health

312 Rosenau Hall | CB#7440

Chapel Hill, NC 27599-7440

mghall@unc.edu

**Supplementary Table 1.** Survey items measuring mediators and response options

| **Construct name and source** | **Item wording** | **Response scale** |
| --- | --- | --- |
| Prompt | The next questions are about different types of beverages. |  |
| Perceived amount of added sugar in SSBs  Adapted from Roberto et al. (2016) | How much **added sugar** do you think is in **regular (non-diet) soda and soft drinks** like the ones above?  **[show image of regular sodas –Pepsi, Sprite]** | 1 (None)  2  3  4  5 (A lot) |
| Perceived amount of added sugar in SSBs  Adapted from Roberto et al. (2016) | How much **added sugar** do you think is in **regular (non-diet) sports drinks** like the ones above?  **[show image of regular Gatorade]** | 1 (None)  2  3  4  5 (A lot) |
| Perceived amount of added sugar in SSBs  Adapted from Roberto et al. (2016) | How much **added sugar** do you think is in **regular (non-diet) flavored waters** like the ones above?  **[show image of regular Vitamin Water]** | 1 (None)  2  3  4  5 (A lot) |
| Perceived amount of added sugar in SSBs  Adapted from Roberto et al. (2016) | How much **added** **sugar** do you think is in **fruit-flavored drinks** **(not 100% juice)** like the ones above?  **[show image of fruit drinks – Sunny D Orange Drink, Ocean Spray Cranberry Juice Cocktail]** | 1 (None)  2  3  4  5 (A lot) |
| Perceived amount of added sugar in SSBs  Adapted from Roberto et al. (2016) | How much **added** **sugar** do you think is in **sweetened pre-packaged teas** like the ones above?  **[show image of teas - sweetened Lipton bottled tea, Snapple peach tea]** | 1 (None)  2  3  4  5 (A lot) |
| Perceived amount of added sugar in SSBs  Adapted from Roberto et al. (2016) | How much **added** **sugar** do you think is in **flavored milks (chocolate, strawberry)** like the ones above?  **[show image of flavored milk]** | 1 (None)  2  3  4  5 (A lot) |
| Prompt | The next questions are about **beverages with added sugar (non-diet).** Examples of beverages with added sugar include regular sodas, fruit-flavored (not 100% juice) drinks, and pre-sweetened coffees and teas. Other examples are regular sports drinks, flavored waters, and energy drinks.  Say how much you agree with each statement below. |  |
| Prompt | Drinking beverages with added sugar every day would increase my [child]’s risk of… |  |
| Perceived likelihood of child having health problems due to SSBs  Adapted from Roberto et al. (2016) | Heart damage | 1 = Not at all  …  5 = A lot |
| Perceived likelihood of child having health problems due to SSBs  Adapted from Roberto et al. (2016) | Type 2 diabetes. | 1 = Not at all  …  5 = A lot |
| Perceived likelihood of child having health problems due to SSBs  Adapted from Roberto et al. (2016) | Having health problems | 1 = Not at all  …  5 = A lot |
| Prompt | **The next questions are also about different types of beverages.** |  |
| Perceived healthfulness of SSBs for child  Adapted from Bollard et al. (2016) | How **unhealthy** or **healthy** is it for your child to drink **[insert category name]**, like the ones above?  [display image of 6 SSB categories]  [repeat question for each category] | 1 = Unhealthy  …  5 = Healthy |
| Appeal of SSBs for child  Adapted from Bollard et al. (2016) | How **unappealing** or **appealing** would your [child] find **[insert category name]**, like the ones above?  [display image of 6 SSB categories]  [repeat question for each category] | 1 = Unappealing  …  5 = Appealing |
| Perceived tastiness of SSBs for child  Adapted from Bollard et al. (2016) | How **not** **tasty** or **tasty** would your [child] find **[insert category name]**, like the ones above?  [display image of 6 SSB categories]  [repeat question for each category] | 1 = Not tasty  …  5 = Tasty |
| Prompt | **The next statements are about the next week (7 days).** |  |
| Intentions to serve SSBs to child  Adapted from Klein et al. (2009) | In the next week, how often do you plan to give your [child] **regular (non-diet) sodas or soft drinks** like the ones above?  **[Show image of sodas]** | 0= Never  1= 1 time per week  2.5= 2-3 times per week  5= 4-6 times per week  7= 1 time per day  14= 2 times per day  21= 3+ times per day |
| Intentions to serve SSBs to child  Adapted from Klein et al. (2009) | In the next week, how often do you plan to give your [child] **regular (non-diet) sports** **drinks** like the ones above?  **[show image of regular Gatorade]** | 0= Never  1= 1 time per week  2.5= 2-3 times per week  5= 4-6 times per week  7= 1 time per day  14= 2 times per day  21= 3+ times per day |
| Intentions to serve SSBs to child  Adapted from Klein et al. (2009) | In the next week, how often do you plan to give your [child] **regular (non-diet) flavored** **waters** like the ones above?  **[show image of regular Vitamin Water]** | 0= Never  1= 1 time per week  2.5= 2-3 times per week  5= 4-6 times per week  7= 1 time per day  14= 2 times per day  21= 3+ times per day |
| Intentions to serve SSBs to child  Adapted from Klein et al. (2009) | In the next week, how often do you plan to give your [child] **fruit-flavored drinks** **(not 100% juice)** like the ones above?  **[show image of fruit drinks]** | 0= Never  1= 1 time per week  2.5= 2-3 times per week  5= 4-6 times per week  7= 1 time per day  14= 2 times per day  21= 3+ times per day |
| Intentions to serve SSBs to child  Adapted from Klein et al. (2009) | In the next week, how often do you plan to give your [child] **sweetened packaged teas** like the ones above?  **[show image of teas]** | 0= Never  1= 1 time per week  2.5= 2-3 times per week  5= 4-6 times per week  7= 1 time per day  14= 2 times per day  21= 3+ times per day |
| Intentions to serve SSBs to child  Adapted from Klein et al. (2009) | In the next week, how often do you plan to give your [child] **flavored milk (chocolate, strawberry)** like the ones above?  **[show image of flavored milks]** | 0= Never  1= 1 time per week  2.5= 2-3 times per week  5= 4-6 times per week  7= 1 time per day  14= 2 times per day  21= 3+ times per day |
| Prompt | **Say how much you disagree or agree with the next statements.** |  |
| Injunctive norms about limiting child’s SSBs  Zoellner et al. (2012) | People who are important to me think my [child] should drink fewer beverages with added sugar each week. | 1 = Strongly disagree  2 = Somewhat disagree  3 = Neither agree nor disagree  4 = Somewhat agree  5 = Strongly agree |
| Injunctive norms about limiting child’s SSBs  Zoellner et al. (2012) | People who are important to me would approve of my [child] drinking fewer beverages with added sugar each week. | 1 = Strongly disagree  2 = Somewhat disagree  3 = Neither agree nor disagree  4 = Somewhat agree  5 = Strongly agree |
| Injunctive norms about limiting child’s SSBs  Zoellner et al. (2012) | People who are important to me want my [child] to drink fewer beverages with added sugar each week. | 1 = Strongly disagree  2 = Somewhat disagree  3 = Neither agree nor disagree  4 = Somewhat agree  5 = Strongly agree |
| Prompt | [intervention] **Above are pictures of labels (stickers) that were on some of the beverages in the store. The next questions are about these labels.**  [Insert image warning labels for experimental group]  [control] **Above is a picture of a label (sticker) that was on some of the beverages in the store. The next questions are about this label.**  [note: items below used “these labels” for intervention group] |  |
| Attention to the labels  Adapted from Brewer et al. (2016) | How much does this label grab your attention? | 1=Not at all  2=Very little  3=Somewhat  4=Quite a bit  5=A great deal |
| Negative emotional reactions  Adapted from Brewer et al. (2016) | How much does this label make you feel **anxious**? | 1=Not at all  2=Very little  3=Somewhat  4=Quite a bit  5=A great deal |
| Negative emotional reactions  Adapted from Brewer et al. (2016) | How much does this label make you feel **scared**? | 1=Not at all  2=Very little  3=Somewhat  4=Quite a bit  5=A great deal |
| Negative emotional reactions  Adapted from Brewer et al. (2016) | How much does this label make you feel **guilty**? | 1=Not at all  2=Very little  3=Somewhat  4=Quite a bit  5=A great deal |
| Thinking about harms of drinking SSBs  Adapted from Fathelrahman et al. (2013) and Hammond et al. (2003) | How much does this label make you think about the health problems caused by drinking beverages with added sugar? | 1=Not at all  2=Very little  3=Somewhat  4=Quite a bit  5=A great deal |
| Anticipated social interactions  Adapted from Hall et al. (2015) | How likely are you to talk about this label with others in the next week? | 1 = Not at all likely  2 = A little likely  3 = Somewhat likely  4 = Very likely  5 = Extremely likely |

**References**

Bollard, T., Maubach, N., Walker, N., & Ni Mhurchu, C. (2016). Effects of plain packaging, warning labels, and taxes on young people's predicted sugar-sweetened beverage preferences: an experimental study. *Int J Behav Nutr Phys Act*, *13*(1), 95. <https://doi.org/10.1186/s12966-016-0421-7>

Brewer, N. T., Hall, M. G., Noar, S. M., Parada Jr, H., Stein-Seroussi, A., Bach, L. E., Hanley, S., & Ribisl, K. M. (2016). Effect of pictorial cigarette pack warnings on changes in smoking behavior: A randomized clinical trial. *JAMA Intern Med*, *176*(7), 905-912. <https://doi.org/10.1001/jamainternmed.2016.2621>

Fathelrahman, A. I., Li, L., Borland, R., Yong, H. H., Omar, M., Awang, R., Sirirassamee, B., Fong, G. T., & Hammond, D. (2013). Stronger pack warnings predict quitting more than weaker ones: finding from the ITC Malaysia and Thailand surveys. *Tobacco Induced Diseases*, *11*(1), 20. <https://doi.org/10.1186/1617-9625-11-20>

Hall, M. G., Peebles, K., Bach, L. E., Noar, S. M., Ribisl, K. M., & Brewer, N. T. (2015). Social interactions sparked by pictorial warnings on cigarette packs. *International Journal of Environmental Research and Public Health*, *12*(10), 13195-13208. <https://doi.org/10.3390/ijerph121013195>

Hammond, D., Fong, G. T., McDonald, P. W., Cameron, R., & Brown, K. S. (2003). Impact of the graphic Canadian warning labels on adult smoking behaviour. *Tobacco Control*, *12*(4), 391-395.

Klein, W. M., Zajac, L. E., & Monin, M. M. (2009). Worry as a moderator of the association between risk perceptions and quitting intentions in young adult and adult smokers. *Annals of Behavioral Medicine*, *38*(3), 256-261. <https://doi.org/10.1007/s12160-009-9143-2>

Roberto, C. A., Wong, D., Musicus, A., & Hammond, D. (2016). The influence of sugar-sweetened beverage health warning labels on parents' choices. *Pediatrics*, *137*(2), e20153185. <https://doi.org/10.1542/peds.2015-3185>

Zoellner, J., Estabrooks, P. A., Davy, B. M., Chen, Y. C., & You, W. (2012). Exploring the theory of planned behavior to explain sugar-sweetened beverage consumption. *J Nutr Educ Behav*, *44*(2), 172-177. <https://doi.org/10.1016/j.jneb.2011.06.010>
