# Supplementary material for: How pictorial warnings change parents’ purchases of sugar-sweetened beverage for their children: mechanisms of impact
Source: Int J Behav Nutr Phys Act. 2023 Jun 23;20:76. doi: 10.1186/s12966-023-01469-3 (PMC10290296; doi:10.1186/s12966-023-01469-3)
Supplement: Supplementary file 2 — Supplementary Material 2 [file 12966_2023_1469_MOESM2_ESM.docx]

September 1, 2022

Richard Rosenkranz, Phd

Editor-in-Chief, *International Journal of Behavioral Nutrition and Physical Activity*

Dear Dr. Rosenkranz:

Please consider the enclosed article entitled “How pictorial warnings change parents’ purchases of sugar-sweetened beverage for their children: Mechanisms of impact” for publication in *International Journal of Behavioral Nutrition and Physical Activity*.

Health warning labels on sugar-sweetened beverages (SSBs) are a promising approach for reducing SSB consumption in children. Prior studies have shown that SSB warnings reduce parents’ purchases of SSBs for their children. Understanding the mechanisms explaining *how* warnings exert their effects could shed light on how to design warnings and other communication efforts to be even more effective.

To our knowledge, our study is the first to examine a wide range of mediators explaining how SSB warnings affect parents’ purchases of SSBs for their children. In this paper, we use secondary data from an RCT with parents (published in *Plos Medicine*, https://doi.org/10.1371/journal.pmed.1003885) to examine mediators of the impact of SSB warnings. We found that health warnings lowered SSB purchases among parents by reducing parents’ perceptions that SSBs are healthy for their children. This mediation pathway is distinct from prior studies among adults buying drinks for themselves, suggesting that parents in particular may respond well to communication campaigns and messages informing them about the poor nutritional quality and health risks of SSBs.

We believe this article fits well with *International Journal of Behavioral Nutrition and Physical Activity*’s focus on behavioral aspects of diet, as well as its focus on theoretical explanations of dietary behavior. This study is methodologically rigorous, using secondary data from a well-powered RCT with a behavioral outcome, conducted in a physical convenience store setting.

We have not previously published the mediation data reported in this paper. As stated in the manuscript, we summarize findings from the main trial paper at the beginning of the results section, demonstrating the impact of warnings (vs. control) on potential mediators. All coauthors have approved the final version of the manuscript. We have no conflicts of interest to disclose. We look forward to your publication decision.

Sincerely,

Marissa G. Hall, PhD (corresponding author)

Assistant Professor

Department of Health Behavior

UNC Gillings School of Global Public Health

312 Rosenau Hall | CB#7440

Chapel Hill, NC 27599-7440

(919) 445-1310

mghall@unc.edu
