# Supplementary material for: How pictorial warnings change parents’ purchases of sugar-sweetened beverage for their children: mechanisms of impact
Source: Int J Behav Nutr Phys Act. 2023 Jun 23;20:76. doi: 10.1186/s12966-023-01469-3 (PMC10290296; doi:10.1186/s12966-023-01469-3)
Supplement: Supplementary file 3 — Supplementary Material 3 [file 12966_2023_1469_MOESM3_ESM.docx]

*Thank you for the opportunity to make minor revisions to our paper #IJBN-D-22-00618 titled “How pictorial warnings change parents’ purchases of sugar-sweetened beverage for their children: Mechanisms of impact” to International Journal of Behavioral Nutrition and Physical Activity (IJBNPA). We thank the reviewer for their excellent feedback and believe we have substantially improved the paper by addressing their comments. Below, our response to each comment is displayed in bold font. Our edits in the paper are marked with highlighted text.*

Reviewer #1: Thanks to the authors for providing this response. There are still some points remaining that need attention:

1.1 Usually a percentage of people (e.g. with 1 or more missing value) is reported.

**In the analysis section, we have clarified that no more than 5% participants had missing data on any of the mediators.**

1.2 Please provide a reference to support this statement in the manuscript, because for as far as I know CONSORT 2010 indicates to report both adjusted and unadjusted results.

**In our prior response to reviewers, a reviewer asked why we did not control for any demographic variables in our analysis. In our first response, we included an explanation of our sensitivity analyses in the response letter but not the paper. However, we have now revised the paper to include an explanation of these analyses. The revised text includes the citation for the CONSORT 2010 guidelines. The revised text is as follows:**

**“We opted not to adjust for covariates in our original analysis based on CONSORT 2010 guidance for RCTs recommending that adjustment is only needed for variables with strong prognostic strength (e.g., stratification variables) (32). However, we performed sensitivity analyses to explore whether adjustment might be warranted for the mediation analyses. We first examined the correlation between key demographics (i.e., age, ethnicity, educational attainment, and annual household income) and the primary outcome of purchasing any SSBs. We planned to re-run the mediation analyses controlling for any characteristics that were significantly correlated with the primary outcome; however, there were no significant correlations, so we did not include these variables in the mediation models.”**

1.3 Also indicate checking (and meeting) assumptions, the same assumptions that underlie (linear) regression are applicable to mediation analyses. (Not) meeting these assumptions might be a reason to still transform or categorize variables that were measured on a continuous scale.

**We evaluated whether assumptions for linear regression were met. We had no violations of multicollinearity given that we conducted simple mediation models with just one mediator per model. We met the assumption of independence as there was no clustering or other non-independence in the data. Many (though not all variables) met the assumptions of homoscedasticity and normal variance distributions. Despite mild violations of these assumptions, we proceeded with using egression for the mediation models given that regression models are quite robust to violations of these assumptions, especially for large sample sizes (Cohen, Cohen, West, & Aiken, 2003). Additionally, as stated in the second paragraph of the analysis section, we bootstrapped the 95% confidence intervals for the indirect effects using the Preacher & Hayes method, which does not assume normality.**

1.4 You do not need PROCESS to calculate c' path, it is the c path, but then additionally adjusting for the mediator variable (to indicate the impact of adjusting for mediating variables and attenuation of the main effect). So, I would propose to also add c' to table 2.

**Although we could manually calculate c’, this value would not be accurate for the binary outcome according to Hayes 2017 (“Introduction to mediation, moderation, and conditional process analysis: A regression-based approach” textbook). Thus, we have not reported c’ for the binary outcome. The c’ for the continuous outcome is reported in Table 3.**

1.5 Please add assumptions that underlie calculation of proportion mediated.

**We have now edited the text to clarify that the PROCESS macro does not calculate the total effect (rather than the proportion mediated). To our knowledge, there are no additional assumptions for calculating the proportion mediated with linear outcomes above and beyond the standard assumptions (as described in 1.3).**
